# Observation of Floquet states in graphene

**DOI:** 10.1038/s41567-025-02889-7

**Published:** 2025-05-06

**Authors:** Marco Merboldt, Michael Schüler, David Schmitt, Jan Philipp Bange, Wiebke Bennecke, Karun Gadge, Klaus Pierz, Hans Werner Schumacher, Davood Momeni, Daniel Steil, Salvatore R. Manmana, Michael A. Sentef, Marcel Reutzel, Stefan Mathias

**Affiliations:** 1 I. Physikalisches Institut, Georg-August-Universität Göttingen, Göttingen, Germany; 2 PSI Center for Scientific Computing, Theory and Data, Paul Scherrer Institute, Villigen PSI, Switzerland; 3 Department of Physics, University of Fribourg, Fribourg, Switzerland; 4 Institut für Theoretische Physik, Georg-August-Universität Göttingen, Göttingen, Germany; 5 Physikalisch-Technische Bundesanstalt, Braunschweig, Germany; 6 Institute for Theoretical Physics and Bremen Center for Computational Materials Science, University of Bremen, Bremen, Germany; 7 Max Planck Institute for the Structure and Dynamics of Matter, Center for Free-Electron Laser Science (CFEL), Hamburg, Germany; 8 International Center for Advanced Studies of Energy Conversion (ICASEC), University of Göttingen, Göttingen, Germany

**Keywords:** Electronic properties and materials, Electronic properties and materials

## Abstract

Floquet engineering—the coherent dressing of matter via time-periodic perturbations—is a mechanism to realize and control emergent phases in materials out of equilibrium. However, its applicability to metallic quantum materials and semimetals such as graphene is an open question. The report of light-induced anomalous Hall effect in graphene remains debated, and a time-resolved photoemission experiment has suggested that Floquet effects might not be realizable in graphene and other semimetals with relatively short decoherence times. Here we provide direct spectroscopic evidence of Floquet effects in graphene through electronic structure measurements. We observe light–matter-dressed Dirac bands by measuring the contribution of Floquet sidebands, Volkov sidebands and their quantum path interference to graphene’s photoemission spectrum. Our results demonstrate that Floquet engineering in graphene is possible, even though ultrafast decoherence processes occur on the timescale of a few tens of femtoseconds. Our approach offers a way to experimentally realize Floquet engineering strategies in metallic and semimetallic systems and for the coherent stabilization of light-induced states with potentially non-trivial topological properties.

## Abstract (web summary)

The feasibility of Floquet engineering in graphene has been called into question due to its fast decoherence processes. Measurements of graphene’s photoemission spectrum now support the generation of Floquet states in this material.

## Main

The field of topological Floquet engineering was pioneered by Oka and Aoki^1^, who proposed that the Haldane model^2^—one of the most paradigmatic models of topology in condensed-matter physics—can be realized in monolayer graphene. On irradiation with circularly polarized light, a dynamical topological anomalous Hall state can be induced, which manifests itself in the formation of Floquet replica of the original Bloch bands and a bandgap opening at the Brillouin zone’s K and K′ points with an inherent change in the Chern number. Such a light-induced topological phase transition can be rationalized within Floquet theory^3–5^, and has been demonstrated experimentally for ultracold fermions in an optical lattice^6^ and photonic crystals^7^.

By now, there is a growing body of work demonstrating elements of Floquet engineering in solid-state materials^8–17^. However, experimental results are mostly limited to insulators, yet the power and versatility of Floquet control is particularly promising for metallic and semimetallic systems, in which a plethora of light-induced phase transitions could be triggered through periodic laser driving^18,19^. Among all materials, the semimetallic two-dimensional Dirac material graphene has proven to be one of the most interesting systems for Floquet engineering, but also a particularly difficult platform for the direct demonstration of Floquet-induced effects. Strongly based on seminal time-resolved photoemission spectroscopy experiments that could not identify Floquet states in graphene^12^, today’s preconception is that the ultrafast decoherence time of only a few tens of femtoseconds^20^ in (semi)metals prevents the generation of Floquet-engineered phases^21,22^. Moreover, the discussion is fuelled by an ultrafast transport measurement that reported a light-induced anomalous Hall effect in graphene^23^, which was interpreted by a combination of Floquet-induced topology and asymmetric photocarrier distributions^24,25^. However, in the interpretation of this work, substantial phenomenological modelling of coupling to a dissipative bath was found to be crucial to identify the Floquet contribution to Hall conductivity^24^. Therefore, the key open question remains whether Floquet engineering can be realized in graphene at all, and to what extent ultrafast dissipation and decoherence might hinder the application of Floquet engineering strategies in metallic systems in general.

In this Article, we use linearly polarized infrared (IR) driving light fields to coherently dress monolayer graphene and then probe the energy–momentum dispersion of the light-dressed band structure with extreme-ultraviolet (EUV) laser pulses generated via high-harmonic generation in a new type of angle-resolved photoemission spectroscopy experiment (ARPES), known as momentum microscopy^26,27^. In a direct comparison of the measured and calculated ARPES maps of a light-dressed band structure, we identify energy- and momentum-resolved fingerprints of Floquet sidebands, Volkov sidebands and their mutual interference. Moreover, we explore the impact of ultrafast dissipation on the formation of Floquet states and their detection in the calculated ARPES maps. Fully in agreement with our experimental results, we show that Floquet states survive the presence of ultrafast scattering, even though light-induced energy gaps become vanishingly small as the quasiparticle lifetime decreases and the spectral function broadens in energy and momentum. Hence, for the first time, our work provides true experimental evidence that the Floquet engineering concept is applicable in (semi)metallic systems. In this way, the study opens up a direct pathway to test the many theoretical proposals of light-induced phase transitions that have remained elusive experimentally so far^5,28–38^.

## Experimental observables for Floquet engineering in graphene

**Fig. 1:**
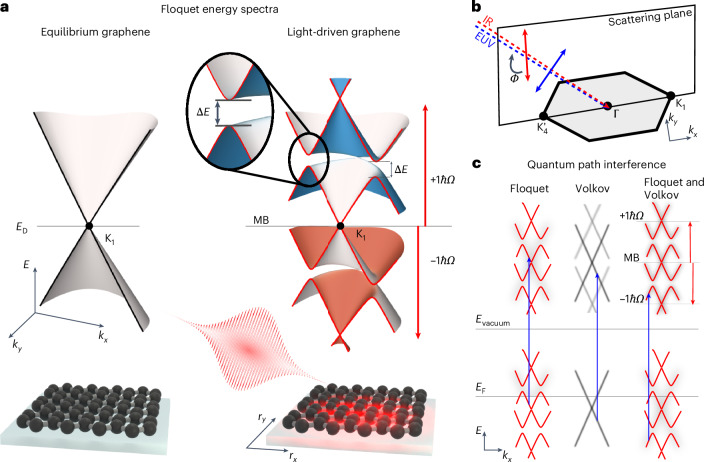
Floquet-engineered modifications to graphene’s electronic structure that are accessible in ARPES experiments. **a**, Energy- and in-plane-momentum-resolved representations of the energy eigenstates of graphene in equilibrium (black) and light-driven graphene (red) in the proximity of the K_1_ point. The characteristics of the Floquet-engineered band structure are (1) the formation of sidebands ±1*ℏΩ* spaced by the driving photon energy from the original MB and (2) the formation of energy gaps Δ*E* in which sidebands of different photon orders cross. **b**, Plane of incidence (scattering plane) of the laser fields is along the ${{\rm{K}}}_{4}^{{\prime} }$–Γ–K_1_ crystal direction. The EUV probe pulses are *p*-polarized and the linear polarization of the IR driving laser pulses is tunable via the polarization angle *Φ*. **c**, Coherent interaction of the IR laser pulses within graphene and with the quasi-free photoelectrons after the photoemission process leads to the formation of Floquet (red) and Volkov (grey) sidebands, respectively. Quantum path interference between the Floquet and Volkov transitions has to be considered, as both states are probed at the same photoelectron energy and in-plane momentum.

**Extended Data Fig. 1:**
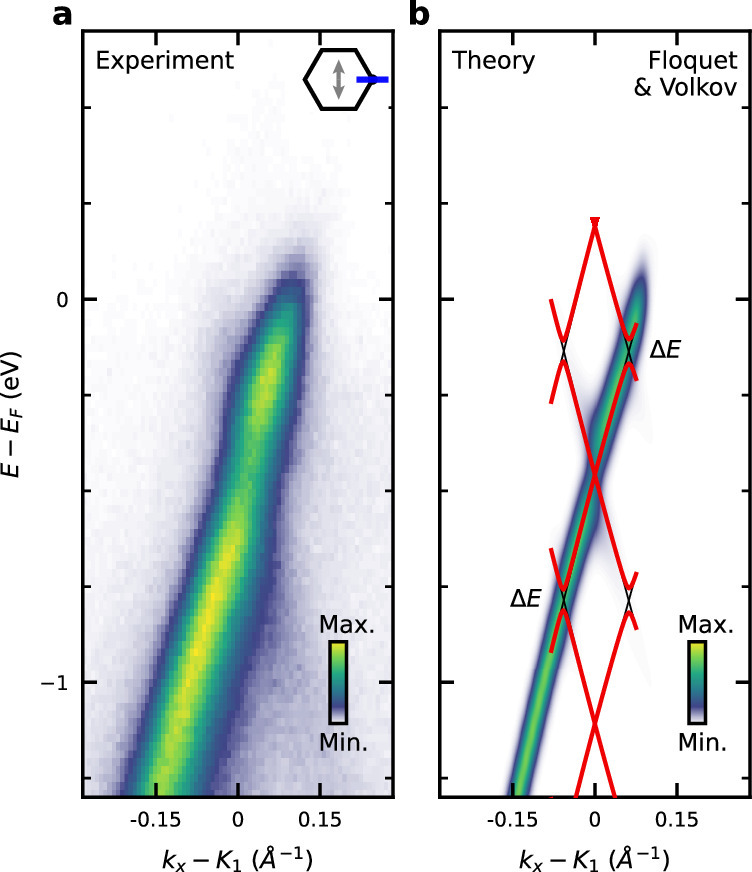
Floquet energy gaps *ΔE* in ARPES experiments with spectrally broad femtosecond laser pulses. **a** Energy- and momentum-resolved photoemission spectrum of light-driven graphene (*s*-polarized) collected in the proximity of the K_1_ point along the *k*_*x*_ momentum-direction (inset). The parameters of the driving light field (the probing light field) are 650 meV, 100 fs, 3 MV/cm (26.5 eV, 20 fs). **b** Floquet energy spectra (red) in the proximity of the K_1_ point calculated for the experimental conditions predict an energy gap of *ΔE* = 70 meV. As a guide to the eye, the black lines indicate the main band and the ± 1st order sidebands neglecting hybridisation. The color-coded data is obtained by calculating an ARPES map for the experimental conditions based on the time-dependent Green’s function formalism. The width of the spectral weight dominantly results from the finite width of the ultrashort EUV probe pulse.

**Extended Data Fig. 2:**
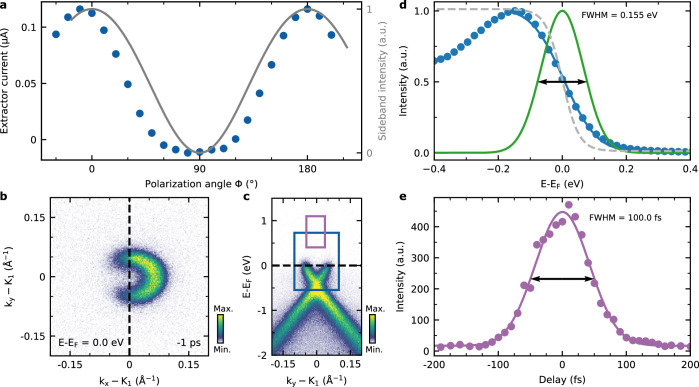
Characterization of the IR pulse polarization *Φ* on the sample, ARPES data of non-driven graphene, and the time- and energy-resolution of the experiment. **a** Irradiation of the graphene sample with IR laser pulses leads to a measurable current between the sample and the extractor, which varies as the pulse’s polarization *Φ* changes from *s* to *p* (data points). As a guide-to-the-eye, the grey line is reproduced from Fig. 2a and shows the calculated Volkov sideband amplitude at the K_1_ point. **b, c** Energy-momentum-resolved (at k_x_-K_1_ = 0 Å^−1^, indicated by dashed line in b)) and momentum-momentum-resolved (E-E_F_ = 0 eV, indicated by dashed line in c)) photoemission data collected when the IR and EUV pulses are far from temporal overlap (*Δt* = − 1 ps). **d** Momentum-filtered energy distribution curve (data points) in the region of interest indicated in panel **c**, blue box. The fit (blue solid line) is carried out with a Fermi-Dirac distribution at 300 K (grey dashed line) convoluted with a Gaussian with a width of 0.155 eV (FWHM, black horizontal line). **e** Quantification of the cross-correlation of the IR driving and the EUV probing laser pulses. The data points are obtained by filtering the photoemission yield from the + 1*ℏΩ* sideband in the magenta 0.7 eV × 0.07 Å^−1^ (k_*x*_) × 0.07 Å^−1^ (k_*y*_) region of interest (both pulses *p*-polarized). The width of the cross-correlation is 100 ± 3 fs (FWHM, black horizontal line).

Figure 1a shows a schematic of the equilibrium (black) and Floquet-engineered (red) electronic structures of graphene (using linearly polarized laser pulses). As discussed in many earlier reports^1,4,5,39^, the light-dressed band structure deviates from its equilibrium counterpart based on two distinct signatures, both of which can be used to unambiguously validate the successful application of Floquet engineering strategies. (1) Floquet theory predicts that higher-order photon-dressed sidebands of the main-band (MB) Dirac cone are formed (Fig. 1a, ±1*ℏΩ* and MB, respectively). (2) Energy bands are gapped where sidebands of different photon orders cross and hybridize in the energy–momentum space (Fig. 1a, Δ*E*). To probe such coherent modifications of graphene’s band structure, the combination of ARPES, particularly momentum microscopy, with a femtosecond pump–probe setup is ideally suited^8,10–12,14,15,17,40^. While an IR laser pulse enables the periodic driving of the system, a time-delayed EUV laser pulse allows to record the energy- and in-plane-momentum-resolved photoemission spectral function. In particular, as broadband ultrashort laser pulses are used in time-resolved ARPES (trARPES) experiments, Floquet energy gaps Δ*E* might not be directly resolvable (Extended Data Fig. 1). Hence, it is more straightforward to study the aforementioned case (1), that is, the photon-dressed sideband formation in momentum space. We opt for this route by using our custom-built photoemission endstation^27,41^ that combines a time-of-flight momentum microscope^26^ with an ultrafast tabletop EUV light source. From the n-doped graphene sample grown on 4H-SiC (electron mobility *μ* ≈ 500–1,000 cm^2^ V^–1^ s^–1^)^42,43^, the momentum microscope facilitates the collection of photoelectrons as a function of energy *E* and both in-plane momenta *k*_*x*_ and *k*_*y*_. In our experimental geometry, the EUV probe (26.5 eV, 20 fs, *p*-polarized) and IR driving (*ℏΩ* = 0.65 eV, 100 fs, 3 MV cm^–1^) pulses impinge nearly co-linearly onto the graphene sample at an oblique angle of incidence of 22° (Extended Data Fig. 2 shows the characterization of pump light polarization and the energy and momentum resolutions of the photoemission experiment). The ${{\rm{K}}}_{4}^{{\prime} }$–Γ–K_1_ crystal direction lies in the scattering plane, and photoelectrons are collected in the proximity of the K_1_ point (Fig. 1b).

## IR polarization dependence of sideband photoemission yield

During the presence of the IR driving laser field, Floquet eigenstates are created and can, in principle, be photoexcited by the EUV laser pulse and measured using ARPES (Fig. 1c, left). However, when ARPES is used for such measurements, it is well known that a competing process can lead to similar photoemission signatures: the coherent interaction of quasi-free photoelectrons with the driving light field leads to the formation of the so-called Volkov sidebands of the main photoemission signature (Fig. 1c, middle)^44,45^. Importantly, these Volkov sidebands look similar to Floquet sidebands in ARPES, but are not an indication for a light-dressed band structure. However, because the Floquet and Volkov transitions are observed at the same final-state energy, quantum path interference effects between both excitation pathways occur and can be observed^10,46^ (Fig. 1c, right). Hence, for the unambiguous identification of Floquet sidebands in ARPES, the experimental challenge lies in the discrimination of Floquet sidebands, Volkov sidebands and their interference pattern.

**Fig. 2:**
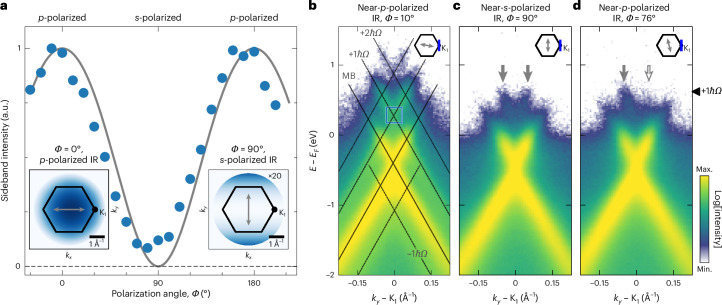
IR polarization dependence of the K_1_ sideband photoemission spectral weight. **a**, IR-polarization-dependent +1*ℏΩ* sideband photoemission yield of K_1_ integrated over the blue region of interest indicated in **b**. The insets show the momentum-resolved Volkov sideband amplitude calculated within the Volkov formalism for *p*-polarized (left) and *s*-polarized (right) IR (blue-shaded colour code; white corresponds to no sideband amplitude; details in Methods and ref. ^40^, calculated using equation (2)). Note that the colour scale for *s*-polarized IR pulses is multiplied by a factor of 20. The corners of the hexagons indicate the momenta of the six K (K′) points of graphene; the K_1_ point is marked with a black dot. The double-headed grey arrows indicate the direction of the in-plane IR electric-field component. From this momentum-resolved analysis of the Volkov formalism, the grey line in the main panel is calculated and shows the Volkov sideband amplitude for the Dirac cone at the K_1_ point for all polarizations *Φ*. **b**–**d**, K_1_ energy- and momentum-resolved photoemission data collected in temporal overlap of the EUV and the IR pulses for *k*_*x*_ ≈ 0 Å^−1^ and *Φ* = 10° (near-*p*-polarized), 90° (*s*-polarized) and 76° (near-*s*-polarized). The vertical grey arrows in **d** highlight the ±*k*_*y*_ asymmetry of the +1*ℏΩ* sideband spectral weight for the near-*s*-polarized IR pulses. The black arrowhead in **d** denotes the centre energy of the momentum maps shown in Fig. 3.

For this task, we make use of the fact that the (*k*_*x*_, *k*_*y*_)-momentum-resolved amplitude of the Volkov sidebands can be controlled by varying the polarization angle *Φ* of the IR field^10,40,46^, and do not yet consider the possible contributions of Floquet states (Methods). For *p*-polarized IR pulses (*Φ* = 0°), the surface-projected electric-field vector **E** is oriented parallel to the scattering plane, and the amplitude of the Volkov sidebands is large for all six K_*i*_ points (Fig. 2a, left inset). By contrast, for *s*-polarized IR pulses (*Φ* = 90°), the surface-projected electric-field vector **E** is oriented perpendicular to the scattering plane, and the Volkov sideband amplitude vanishes for all momenta along the ${{\rm{K}}}_{4}^{{\prime} }$–Γ–K_1_ crystal direction (Fig. 2a, right inset). On the basis of these calculations, Fig. 2a shows the plot of the calculated polarization dependence of the Volkov sideband amplitude at the momentum of the K_1_ point (grey line). The Volkov sideband amplitude is maximized for *p*-polarized light and vanishes for *s*-polarized light. Hence, if it is possible to observe sideband photoemission spectral weight in the proximity of the K_1_ point for *s*-polarized IR light, where the Volkov sideband amplitude must be zero, the result would be indicative for the formation of Floquet states.

Figure 2b–d shows the energy- and momentum-resolved photoemission data collected in temporal overlap of the EUV and IR laser pulses; the polarization of the IR pulses is *Φ* = 10° (near-*p* polarization), 90° (*s* polarization) and 76° (near-*s* polarization). Starting with the case of near-*p* polarization, we can clearly identify the main Dirac cone (MB), as well as sidebands spaced by –1*ℏΩ*, +1*ℏΩ* and +2*ℏΩ* (Fig. 2b). To evaluate the impact of IR polarization on the sideband photoemission yield, we systematically evaluate the data in a 0.064 Å^−1^ × 0.064 Å^−1^ × 0.22 eV region of interest at the +1*ℏΩ* sideband (Fig. 2b, blue box). For all polarizations *Φ*, we detect a spectral weight originating from the +1*ℏΩ* sideband (Fig. 2a, data points). In particular, even for the case of *s*-polarized IR pulses, we still identify the spectral weight of the +1*ℏΩ* sideband (Fig. 2c). This observation is in stark contrast to the expected fingerprints in the hypothetical case of Volkov states only, which should not lead to a finite +1*ℏΩ* sideband photoemission yield for the *s*-polarized IR pulses (Fig. 2a, grey line).

The natural follow-up question is whether the +1*ℏΩ* sideband photoemission signal collected for *s*-polarized IR light already constitutes unambiguous proof for the existence of Floquet states (Fig. 2c), especially since such a signature could not be identified in a previous ARPES experiment^12^. For instance, it might be possible that the linear polarization of the IR pulses is not sufficiently pure, which would also lead to a finite Volkov sideband intensity. Note that we exclude this scenario in our case, because we use a polarization extinction ratio that is better than 200:1. Nevertheless, an additional hallmark that supports the successful generation of Floquet states seems necessary. Reviewing Fig. 2b–d, we find another very strong signature in the energy–momentum-resolved photoemission spectra that is incompatible with the Volkov picture alone: although the near-*p*-polarized and near-*s*-polarized measurements show symmetric +1*ℏΩ* sideband spectral weight in the ±*k*_*y*_ momentum direction (Fig. 2b,c), for *Φ* = 76°, the measurement exhibits a striking ±*k*_*y*_ asymmetry between the two sides of the Dirac cone (Fig. 2d, vertical arrows). In the following, we will show that such strongly asymmetric photoemission signatures of the observed sidebands cannot be explained within the Volkov or the Floquet picture alone, but must be a result of the quantum path interference of Volkov and Floquet states.

## Verification of Floquet states in graphene

**Fig. 3:**
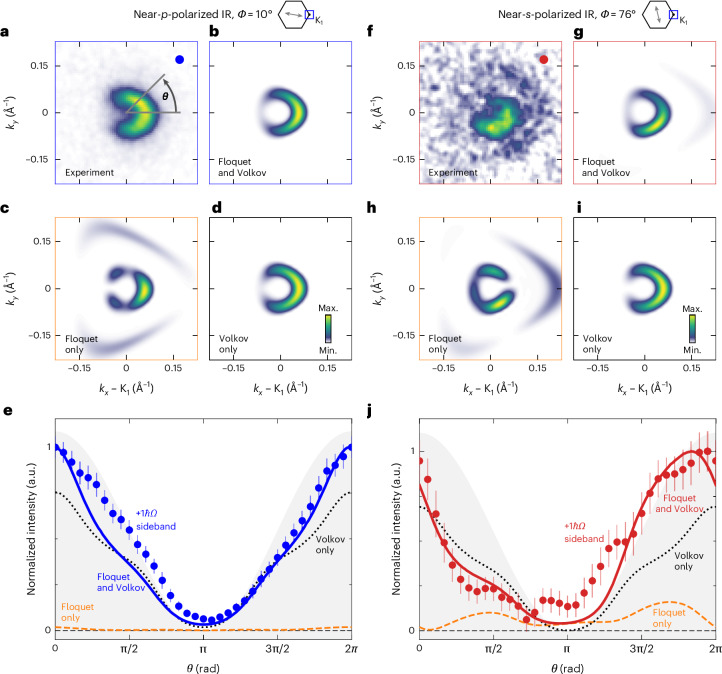
Sideband photoemission momentum fingerprints and quantum path interference between Floquet and Volkov transitions for the K_1_ cone. **a**–**d**,**f**–**i**, Measured (**a**) and calculated (**b**–**d**) +1*ℏΩ* sideband momentum fingerprints for near-*p*-polarized (*Φ* = 10°), and the measured (**f**) and calculated (**g**–**i**) sideband momentum fingerprints for near-*s*-polarized (*Φ* = 76°) IR pulses (*E* – *E*_F_ = 0.63 eV, Δ*t* = 0 fs; +1*ℏΩ-*labelled arrowhead in Fig. 2d). The theory maps contain the coherent sum of Floquet and Volkov transitions (**b** and **g**), Floquet transitions only (**c** and **h**) and Volkov transitions only (**d** and **i**). **e**,**j**, Momentum fingerprint of the +1*ℏΩ* sideband spectral weight is parameterized with angle *Θ* (**a**).The data points are obtained by integrating background-corrected photoemission counts for each 0.349 rad opening angle *Θ*. The error is determined by the standard deviation of the total counts per segment caused by the uncertainty in the position of the K_1_ point. In addition, we added ±10% of the background counts to the error bars (Methods). **e**, For near-*p*-polarized IR pulses, the measured photoemission momentum fingerprint of the +1*ℏΩ* sideband (blue circles) qualitatively mirrors the *Θ* dependence of the dark corridor (grey area), and is well described by the calculations that consider Floquet and Volkov transitions (blue line). **j**, In the case of near-*s*-polarized IR pulses, the measured photoemission momentum fingerprint of the +1*ℏΩ* sideband (red circles) is qualitatively different to the *Θ* dependence expected from the dark corridor, and shows a distinct asymmetry with a minimum and maximum for *Θ* < π and *Θ* > π, respectively. Neither the Volkov-only (black dotted line) nor the Floquet-only (orange dashed) calculations predict this strong asymmetry. Agreement between experiment and theory can only be found if quantum path interference processes between Floquet and Volkov transitions are considered (red line).

**Extended Data Fig. 3:**
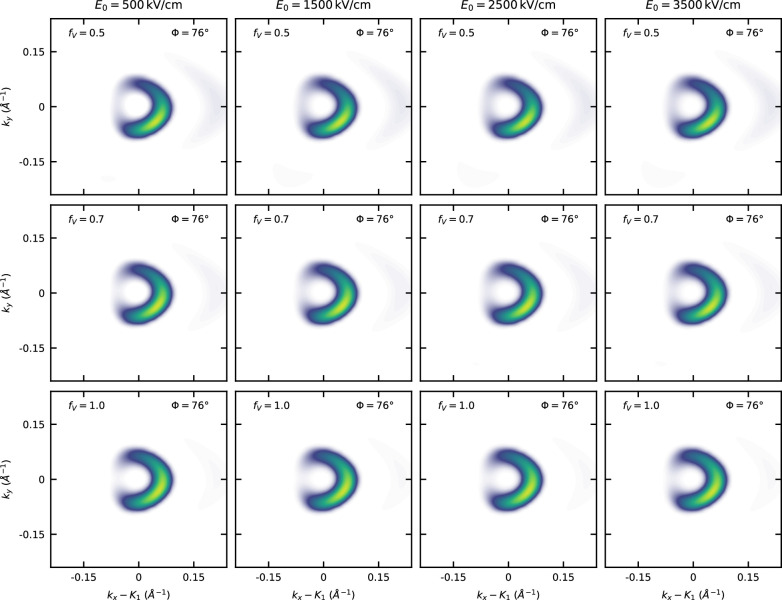
Overview of the calculated Floquet and Volkov + 1*ℏΩ* sideband momentum fingerprint at *Φ* = 76^∘^ as a function of the vacuum electric field strength *E*_0_ and the factor *f*_*V*_. From top to bottom, the parameter *f*_*V*_ is varied from 0.5 to 1. From left to right, the vacuum electric field strength *E*_0_ is varied from 0.5 MV/cm to 3.5 MV/cm. The Floquet scaling factor is set to *f*_*F*_ = 0.5 for all shown momentum maps. For all figures in the main text, we choose *f*_*F*_ = *f*_*V*_ = 0.5 and *E*_0_ = 3 MV/cm.

To investigate the ±*k*_*y*_ momentum asymmetry in more detail, we make use of the full momentum-resolved data collection capability of our photoemission endstation and generate (*k*_*x*_, *k*_*y*_)-resolved photoemission maps at the energy of the +1*ℏΩ* sideband (Fig. 3a,f). Although the momentum map of the near-*p*-polarized case (*Φ* = 10°) shows the well-known horseshoe-like spectral weight distribution in the sideband that originates from the dark corridor effect^47^ (Fig. 3a), in the case of near-*s*-polarized IR pulses (*Φ* = 76°), the spectral weight distribution is dominated by the strong ±*k*_*y*_ asymmetry (Fig. 3f) that was already observed in the energy–momentum-resolved data shown in Fig. 2d. To show that this asymmetry provides unambiguous evidence for Floquet states in graphene, we compare our experimental data with ARPES momentum maps calculated within the time-dependent non-equilibrium Green’s function formalism (td-NEGF, Methods and Extended Data Fig. 3). The calculations are performed such that the spectral weight in the momentum maps can contain contributions of the coherent sum of Floquet and Volkov sidebands (Fig. 3b,g), Floquet sidebands only (Fig. 3c,h) or Volkov sidebands only (Fig. 3d,i), as labelled in the respective momentum maps (Fig. 3). Intriguingly, already from the visual inspection of the momentum maps, it is obvious that the experimental ±*k*_*y*_ momentum asymmetry for the near-*s*-polarized IR pulses (Fig. 3f) can only be reproduced by the calculations that consider constructive and destructive quantum path interference processes between Floquet and Volkov transitions (Fig. 3g). Neither the calculated momentum dependence of the pure Floquet (Fig. 3h) nor that of the pure Volkov (Fig. 3i) transitions can reproduce the experimentally observed asymmetry.

We evaluate the momentum-dependent spectral weight distribution of the +1*ℏΩ* sideband in experiment and theory by integrating the sideband signal in segments around the K_1_ point parameterized with angle *Θ* (Fig. 3a). For near-*p*-polarized IR pulses (Fig. 3e), we make two important observations. First, we find that the experimental data (blue circles) follows the *Θ* dependence expected from the dark corridor effect^47^ (grey area), indicating that the +1*ℏΩ* sideband spectral weight is a near-perfect replica of the non-driven MB (Extended Data Fig. 2c). Second, the experimental *Θ* dependence is well described by the calculations that include the coherent contribution of Floquet and Volkov states (blue solid line). However, by comparing the *Θ*-dependent spectral weight of the Volkov-only (black dotted line) and Floquet-only (orange dashed line) calculations, it is obvious that mainly Volkov transitions contribute to the measured +1*ℏΩ* sideband intensity, as expected for near-*p*-polarized driving.

Next, we repeat the same evaluation for the +1*ℏΩ* sideband momentum pattern for the case of near-*s*-polarized IR pulses (*Φ* = 76°). In Fig. 3j, it is directly clear that the sideband’s photoemission spectral weight (red circles) deviates from the pure cosine-like *Θ* dependence and, thus, does not follow the periodicity of graphene’s dark corridor (grey area). To verify the contribution of Floquet states at *Φ* = 76° IR driving, Fig. 3j shows the *Θ* dependence of the spectral weight of the calculated momentum maps for Floquet-only calculation (orange line), Volkov-only calculation (black line) and the case of photoemission quantum path interference of Floquet and Volkov states (red line). In particular, the *Θ* dependence of the Volkov-only solution is close to symmetric for *Θ* ≶ π, as found for the case of near-*p*-polarized IR driving, but in contrast to the experimental data of the *Φ* = 76° case. Hence, the measured momentum-asymmetric +1*ℏΩ* sideband spectral weight distribution cannot be described within the Volkov formalism alone. Likewise, the calculated Floquet-only momentum dependence is close to symmetric for *Θ* ≶ π and, therefore, does not reproduce our experimental observations either. However, in the case that constructive and destructive quantum path interference processes of Floquet and Volkov transitions are considered, our calculations clearly reproduce the experimentally observed asymmetry (red line). In other words, the strong asymmetric-momentum fingerprint of the +1*ℏΩ* sideband intensity can only be observed if the Floquet and Volkov states are detected, thereby directly verifying the experimental realization of Floquet effects in graphene.

## Quantum path interference of Floquet and Volkov states

**Fig. 4:**
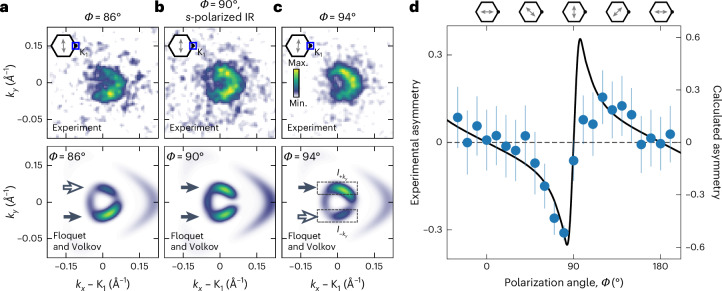
Control of quantum path interference between Floquet and Volkov states via IR pulse polarization *Φ* for the K_1_ cone. **a**–**c**, The top and bottom rows of panels show the measured and calculated photoemission momentum maps of the +1*ℏΩ* sideband, respectively (*E* – *E*_F_ = 0.63 eV, Δ*t* = 0 fs). As the polarization of the IR pulses (double-headed arrows in the inset) is rotated from *Φ* = 90° – 4° = 86° (**a**) to *Φ* = 90° (**b**) and *Φ* = 90° + 4° = 94° (**c**), the spectral weight becomes more intense for –*k*_*y*_, similarly intense for ±*k*_*y*_ and more intense for +*k*_*y*_ (horizontal arrows in the bottom panels). **d**, Systematic evaluation of the *Φ*-dependent asymmetry $A=({I}_{+{{\rm{k}}}_{{\rm{y}}}}-{I}_{-{{\rm{k}}}_{{\rm{y}}}})/({I}_{+{{\rm{k}}}_{{\rm{y}}}}+{I}_{-{{\rm{k}}}_{{\rm{y}}}})$ (regions of interest are indicated in **c**). The data points and the black line are extracted from experiment and theory, respectively (Methods). The asymmetry *A* changes sign when the surface-projected electric-field vector (double-headed arrow) is rotated through *Φ* = 0°, *Φ* = 90° and *Φ* = 180°. The error is determined by the standard deviation of the total counts per segment caused by the uncertainty in the position of the K_1_ point (Methods).

Our results indicate that the momentum-resolved sideband photoemission spectral weight is dependent on the relative phase of the Floquet and Volkov transitions contributing to the quantum path interference conditions. As initially discussed for Bloch bands in the proximity of the Γ point^46^ and recently extended to bands at the K point^48^, the phase of the Floquet amplitude is determined by the projection of the IR field onto momentum **k**, whereas the Volkov phase exhibits a much weaker dependence. Therefore, as in an interferometer in which the phase of one channel can be controlled, it must be possible to flip the asymmetric-momentum fingerprint of the +1*ℏΩ* sideband by controlling the polarization angle around *Φ* = 90° (*s* polarization). In Fig. 4a–c, we show the measured (top row) and calculated (bottom row) momentum maps of the +1*ℏΩ* sideband for *Φ* = 90° (Fig. 4b) and *Φ* = 90° ∓ 4° (Fig. 4a,c). In the case of *s*-polarized IR pulses, the surface-projected electric-field vector is oriented perpendicular to the scattering plane and the quantum path interference conditions are symmetric in the +*k*_*y*_ and –*k*_*y*_ directions (Fig. 4b). By contrast, if *Φ* ≠ 90°, the surface-projected electric-field vector and the scattering plane are not perpendicular anymore with respect to each other, and quantum path interference leads to an asymmetric spectral weight for ±*k*_*y*_ (Fig. 4a,c). In particular, the asymmetry flips for angles ≶90°, as expected from theory. Finally, Fig. 4d shows the systematic evaluation of the momentum asymmetry $A=({I}_{+{{\rm{k}}}_{{\rm{y}}}}-{I}_{-{{\rm{k}}}_{{\rm{y}}}})/({I}_{+{{\rm{k}}}_{{\rm{y}}}}+{I}_{-{{\rm{k}}}_{{\rm{y}}}})$ as a function of the IR pulse polarization angle *Φ*. In agreement between experiment (blue dots) and theory (black line), we find that the asymmetry *A* increases from the *p*-polarized case (*Φ* = 0° and *Φ* = 180°) to the *s*-polarized case (*Φ* = 90°). Close to *s* polarization (*Φ* = 90°), *A* flips and is the most sensitive to changes in polarization, as both Floquet and Volkov sideband contributions have comparable amplitudes.

## Floquet effects and the impact of ultrafast scattering

**Fig. 5:**
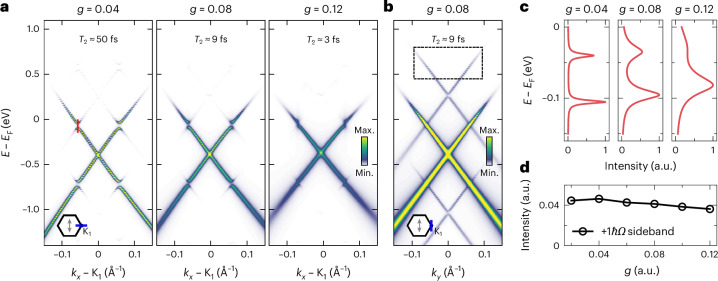
Floquet effects and the impact of ultrafast scattering. **a**, Floquet spectral function *A*(**k**, *ω*) calculated for momenta close to the K_1_ point in the *k*_*x*_ direction for increasing strength of the electron–phonon coupling *g*. **b**, Example of a calculated spectrum for the *k*_*y*_ direction. **c**,**d**, Momentum-filtered energy distribution curves showing the Floquet-induced gap (indicated by the red vertical line in the leftmost panel in **a**) (**c**) and total sideband spectral weight in the *k*_*y*_ direction (indicated by black dashed box in **b**) for increasing *g* (**d**). Even though an increasing *g* leads to an energetic broadening, the total spectral weight of the Floquet sidebands remains close to constant.

Finally, one of the most discussed effects that might hinder the creation of Floquet states in a material is the impact of ultrafast scattering and decoherence^12,21^. Importantly, in contrast to the trARPES experiments reported in ref. ^12^, our experiment proves the existence of Floquet effects in graphene in the presence of ultrafast scattering^43^ and for a sample with similar electron mobility as used in ref. ^12^ (500–1,000 cm^2^ V^–1^ s^–1^). Therefore, to shed light on the influence of ultrafast decoherence, we carried out additional theoretical simulations that use the td-NEGF formalism and include a self-energy that incorporates dissipation and dephasing effects through electron–phonon interaction (Methods). We aim to compare the decay of coherence in the microscopic electron–phonon model to the phenomenological dephasing time *T*_2_ as determined experimentally (${T}_{2}^{\,\exp }$ ≈ 20 fs)^20^. Although a one-to-one correspondence can only be an estimate due to the details of the scattering processes, values of *g* > 0.06 are roughly equivalent to *T*_2_ < 20 fs. For coupling strength *g* > 0.08, *T*_2_ is already shorter than 10 fs. In Fig. 5, we present the corresponding spectral functions *A*(**k**, *ω*) for cuts through the K_1_ point. For small electron–phonon coupling, the energy–momentum spectra are very sharp and the light-induced energy gaps can clearly be resolved in the momentum-filtered energy distribution curves (Fig. 5a,c, *g* = 0.04 and *T*_2_ ≈ 50 fs). Increasing *g* gives rise to large intrinsic broadening that eventually obscures any light-induced gap openings, which is consistent with the phenomenological dephasing model used earlier^12^. Nevertheless, our simulations show that the Floquet sidebands remain visible (Fig. 5d), in agreement with our experimental result.

## Conclusions and outlook

We directly demonstrate the successful generation of Floquet states in graphene. Our work unambiguously extends the application of Floquet engineering strategies beyond insulators^8,10,15^ and semiconductors^12,14,16,17^ in which energy dissipation and decoherence processes are slow. Instead, our work shows that coherent materials engineering is possible even in (semi)metallic materials with decoherence times as short as a few tens of femtoseconds. Although decoherence processes lead to a broadening of the energetic linewidth measured in the ARPES experiment, as shown in other work^12^, they have no major impact on the formation of Floquet states in general. In conclusion, our work motivates the application of Floquet engineering strategies to metallic systems^18,19^ and, consequently, opens up a plethora of opportunities for coherent materials engineering^5,28–38^. A particularly exciting opportunity opened up by our direct demonstration of Floquet engineering in a graphitic material is its application to moiré heterostructures with metallic bands and complex phase diagrams, sparked by the groundbreaking discovery of superconductivity in magic-angle twisted bilayer graphene^49^.

## Methods

### Floquet energy gaps in trARPES experiments

In the section ‘Experimental observables for Floquet engineering in graphene’ of the main text, we highlight two options to observe a Floquet-engineered band structure in an ARPES experiment. Option (2) is the identification of light-induced energy gaps in which Floquet bands of different photon orders cross (Fig. 1a, Δ*E*). For this, Extended Data Fig. 1a shows the energy–momentum-resolved photoemission data taken along the *k*_*x*_ momentum direction for *s*-polarized IR pulses (K_1_ point, *Φ* = 90°), that is, the momentum direction in which Floquet energy gaps are expected^5,10,12^. However, the data show no clear indication for energy gaps.

To verify whether this result is caused by the limited energy resolution in our ultrafast ARPES experiment with spectrally broad laser pulses, we compare the experimental data with our model calculations. For this, we first calculate the energy–momentum dispersion of the Floquet eigenenergies (Extended Data Fig. 1b, red lines). For the 650-meV IR pulses with a vacuum electric-field strength of 3 MV cm^–1^, we extract a Floquet energy gap of Δ*E* = 70 meV. Second, we calculate an ARPES spectrum of the light-driven band structure within the non-equilibrium Green’s function formalism (Extended Data Fig. 1b, colour-coded data). The ARPES spectrum now intrinsically shows a distinct energy broadening, which is mainly caused by the spectrally broad pump and probe laser pulses. We note that the linewidth of the calculated ARPES signature is much narrower than found in our experimental results, indicating that even other broadening effects contribute to the experimental broadening (155 meV; Extended Data Fig. 2d), which are not captured in the model. Nevertheless, even in the calculated ARPES spectrum, it is not straightforwardly possible to identify a clear spectroscopic signature of an energy gap. In consequence, for the parameters of the driving light field that are currently accessible with our setup, we conclude that the energy resolution of the time-resolved momentum microscopy experiment is simply not sufficient to directly resolve the spectroscopic signatures of energy gaps.

### Experimental setup and sample preparation

The time-resolved momentum microscopy experiments have been performed using our custom-built photoemission endstation that combines a time-of-flight momentum microscope^26^ (Surface Concept) and a 300-W fibre laser system (Active Fiber Systems)^27^. The laser is operated at 500 kHz and drives a tabletop high-harmonic-generation beamline and an optical parametric amplifier (LIGHT CONVERSION)^27,41^. The high-harmonic-generation beamline is operated with 5-W, 50-fs, 515-nm pulses focused into argon gas, and the 11th harmonic (26.5 eV, *p*-polarized) is selected with a pair of EUV multilayer mirrors. The optical parametric amplifier is operated at 40-W input power (1,030 nm, 220 fs) and generates the 210-mW (at sample), 100-fs, 0.65-eV IR driving laser pulses. The polarization angle *Φ* of the IR pulses is varied with an achromatic half-wave plate (B. Halle, 700–2,500 nm). The extinction ratio of the driving laser pulses is determined to be *T*_p_:*T*_s_ > 200:1. The polarization *Φ* of the IR pulses on the sample is determined by monitoring the extractor current as a function of the IR polarization angle (Extended Data Fig. 2a) and the direct comparison of the sideband momentum fingerprints in experiment and theory (Fig. 4).

All experiments are performed at room temperature on an n-doped graphene sample grown on 4H-SiC^42,43^. In an ultrahigh vacuum, the graphene sample was annealed for 1 h at 450 °C. For all the experiments, the microscope was aligned such that a momentum area with a diameter of 1.3 Å^−1^ centred on the K_1_ point is projected onto the detector^27^. In addition, the multidimensional photoemission data shown throughout the text are corrected for distortions in energy and momentum^50–52^. The vacuum electric-field strength of the IR pulses is approximated to 3 MV cm^–1^ with an estimated 1/*e*^2^ diameter of the IR beam of 300 μm × 250 μm and a peak fluence of 1.23 mJ cm^–2^. Precise control of the polarization angle of the linearly polarized IR driving pulses was achieved by using a motorized rotation mount (Thorlabs).

### Time and energy resolutions of the photoemission experiment

We quantify the energy resolution of the momentum microscopy experiment by fitting a momentum-filtered energy distribution curve with a Fermi–Dirac distribution broadened by a Gaussian distribution (Extended Data Fig. 2b–d). The data are taken from the measurement at a delay of –1 ps, that is, the IR and EUV pulses are on the sample but not in temporal overlap (Extended Data Fig. 2b,c). Keeping the temperature of the Fermi–Dirac distribution fixed to 300 K, the fit yields a Gaussian width of 155 ± 47 meV (Extended Data Fig. 2d). The Gaussian width then describes the energy resolution of our experiment and contains contributions from the spectral width of the laser pulses and the energy resolution of the momentum microscope^27^.

In Extended Data Fig. 2e, we evaluate the pump–probe delay dependence of the +1*ℏΩ* sideband photoemission signal to extract the cross-correlation of the IR and EUV beams to 100 ± 3 fs (full-width at half-maximum). With a pulse duration of the EUV pulses of 20 ± 5 fs (refs. ^27,53^), we extract the pulse duration of the IR pulse to be 98 ± 4 fs. This translates to a Fourier-limited spectral width of 27 ± 1 meV and 124 ± 30 meV for the IR and EUV laser pulses, respectively.

### Data collection and data processing

For the IR polarization-dependent +1*ℏΩ* sideband yield (Fig. 2a), the polarization angle *Φ* was varied in 5° steps. At each *Φ*, the photoemission signal was integrated for 60 s. The photoelectron counts in the region of interest (Fig. 2b, blue box) are integrated. The size of the region of interest is 0.064 Å^−1^ × 0.064 Å^−1^ × 0.22 eV.

Detailed evaluation of the momentum-dependent spectral weight distribution of the +1*ℏΩ* sideband in Fig. 3 in experiment and theory was done by integrating the sideband signal in segments around the K_1_ point parameterized with angle *Θ* (Fig. 3a). Each segment contains an opening angle of 0.349 rad (20°). Between consecutive segments, the angle *Θ* was varied by 0.175 rad (10°). This ensures that the relative intensities between Floquet, Volkov and their coherent sum are conserved. The experimental data points are shown after a background subtraction. The background was determined by integrating the photoemission counts between 0.126 Å^−1^ < ∣*k*∣ < 0.221 Å^−1^ around K_1_. The constant background was subtracted from each data point. The experimental errors are determined by varying the centre position of the segments in a radius in momentum space of 1 binned pixel of the detector in all directions, which corresponds to the main uncertainty in the data analysis procedure. Additionally, 10% of the subtracted background was assumed to contribute to the uncertainty of the data points.

The polarization-dependent asymmetry signal in Fig. 4d was calculated by extracting the photoemission counts in the regions of interest ${I}_{\pm {k}_{y}}$; the example indicated in Fig. 4c shows the upper and lower parts of the +1*ℏΩ* sideband, respectively. The experimental error is determined by varying the centre position of the regions of interest in a radius in momentum space of 1 binned pixel of the detector in all directions, which corresponds to the main uncertainty in the data analysis procedure.

### Momentum- and polarization-dependent Volkov sideband amplitude

In the following, we briefly describe the polarization and momentum dependencies of the Volkov sideband yield, as discussed in the main text and shown in Fig. 2a (insets and grey line). Details on this analysis can be found elsewhere^40,44,46,54^, and we follow the earlier work in ref. ^40^. The photoelectron momentum distribution of the first-order Volkov sideband is given by

1 $${I}_{1}\left({k}_{xy},{\theta }_{k},{k}_{z}\right) \sim {I}_{0}^{{\prime} }\left({k}_{xy},{\theta }_{k},{k}_{z}\right) \times | {\alpha }_{1}{| }^{2},$$

where ${I}_{0}^{{\prime} }\left({k}_{xy},{\theta }_{k},{k}_{z}\right)$ is the photoemission yield of the undriven system, and |*α*_1_|^2^ is the Volkov sideband amplitude. In the electron scattering description^44,46,54^, the *α* parameter can be expressed as

2 $$\alpha \sim \left(\frac{e}{{m}_{e}{\varOmega }^{2}}({E}_{xy}{k}_{xy}\cos ({\theta }_{k}-{\theta }_{E})+{E}_{z}{k}_{z})\right).$$

Here the in-plane electric-field components and the in-plane momentum components are expressed in polar coordinates, that is, *θ*_*k*_ = tan^–1^(*k*_*y*_/*k*_*x*_) (measured from the Γ point) and *θ*_*E*_ = tan^–1^(*E*_*y*_/*E*_*x*_) (ref. ^40^). Moreover, *e*, *m*_e_ and *Ω* are the elementary charge, elementary mass and driving light frequency, respectively. In the insets in Fig. 2a, the momentum-dependent distribution of |*α*_1_|^2^, which describes the Volkov amplitude, is plotted for *s*- and *p*-polarized IR pulses in our experimental geometry (Fig. 1b and ref. ^40^).

### Details of calculations

#### Experimental parameters that enter the calculations

For the determination of the experimental parameters that enter our simulations, we start with the measured vacuum-field strength of *E*_0_ ≈ 3 MV cm^–1^ (see the ‘Floquet energy gaps in trARPES experiments’ section). Further, we need to adjust the electric-field strength at the surface and inside the graphene, because the interface between graphene and vacuum is not a sharp interface as assumed for the Fresnel equations. To do so, we introduce scaling factors *f*_V_ and *f*_F_ for the Volkov and Floquet field strengths at the surface and inside the graphene, respectively.

The electric-field strength that generates Volkov sidebands at the surface is then

3 $${{\bf{E}}}_{{\rm{eff}}}(\varPhi )={f}_{{\rm{V}}}\left({{\bf{E}}}_{{\rm{in}}}(\varPhi )+{{\bf{E}}}_{{\rm{r}}}(\varPhi )\right),$$

where **E**_in_(*Φ*) (**E**_r_(*Φ*)) is the amplitude of the incoming (reflected) pump field for a given polarization angle *Φ*. The reflected field is computed by decomposing **E**_r_ into *s* and *p* components and using the Fresnel equations. In direct comparison of our calculations with the experimental results, we fix *f*_V_ = 0.5. We note that this value is in good agreement with a study on dielectric screening on the atomic-length scale^55^.

The local effective field driving the electrons inside the graphene sample is also modified by the screening. To account for the screening of the field inside the graphene, we interpolate the fields by approximating the pump field **E**_pump_ by

4 $${{\bf{E}}}_{{\rm{pump}}}(\varPhi )={f}_{{\rm{F}}}\left({{\bf{E}}}_{{\rm{in}}}(\varPhi )+{{\bf{E}}}_{{\rm{r}}}(\varPhi )\right)+(1-{f}_{{\rm{F}}}){{\bf{E}}}_{{\rm{t}}}(\varPhi ).$$

Here **E**_t_(*Φ*) is the transmitted-field amplitude. We chose *f*_F_ = 0.5, assuming that the effective electric field **E**_pump_ interpolates between the field outside and inside the material. We note again that this value is in good agreement with ref. ^55^. Finally, we stress that although there is a considerable uncertainty in *E*_0_, the momentum-space signatures of the Floquet–Volkov interference (as discussed in the main text) are unaffected over a large parameter range (Extended Data Fig. 3).

From the electric-field amplitudes, we also obtain the time-dependent vector potentials **A**_eff_(*t*) and **A**_pump_(*t*), which enter the calculation of the trARPES signals.

#### Time-dependent dynamics

The pump-induced dynamics are described by solving the equation of motion for the density matrix:

5 $$\frac{{\rm{d}}}{{\rm{d}}t}{\bf{\rho }}({\bf{k}},t)=-{\rm{i}}[{\bf{H}}({\bf{k}},t),{\bf{\rho }}({\bf{k}},t)]+D[{\bf{\rho }}({\bf{k}},t)]\,.$$

Here *D*[**ρ**(**k**, *t*)] denotes a scattering term incorporating pure dephasing dynamics with a decoherence time of *T*_2_ = 20 fs. The decoherence of the off-diagonal elements of **ρ**(**k**, *t*) is defined with respect to the instantaneous Hamiltonian **H**(**k**, *t*) (ref. ^56^). The Hamiltonian is formulated in the velocity gauge, which allows us to consistently compute the time-resolved photoemission signal and retaining gauge invariance^57^. In the velocity gauge,

6 $${H}_{\alpha {\alpha }^{{\prime} }}({\bf{k}},t)={\varepsilon }_{\alpha }({\bf{k}}){\delta }_{\alpha {\alpha }^{{\prime} }}-{{\bf{A}}}_{{\rm{pump}}}(t)\cdot {{\bf{v}}}_{\alpha {\alpha }^{{\prime} }}({\bf{k}})+\frac{1}{2}{{\bf{A}}}_{{\rm{pump}}}{(t)}^{2}.$$

We have computed the electronic band structure *ε*_*α*_(**k**) and the velocity matrix elements ${{\bf{v}}}_{\alpha {\alpha }^{{\prime} }}({\bf{k}})=\langle {\psi }_{{\bf{k}}\alpha }| \hat{{\bf{p}}}| {\psi }_{{\bf{k}}{\alpha }^{{\prime} }}\rangle$ using our custom-built all-electron density functional theory code. Consistency with the standard codes QUANTUM ESPRESSO and WANNIER90 has been checked. We included the two Dirac-like bands *α* = 1 and *α* = 2 in equations (5) and (6).

The pump pulse is parameterized by a Gaussian pulse as

7 $${{\bf{A}}}_{{\rm{pump}}}(t)=\frac{1}{\varOmega }S(t){\rm{Re}}\left[{\rm{i}}{{\bf{E}}}_{{\rm{pump}}}{{\rm{e}}}^{-{\rm{i}}\varOmega t}\right],$$

where *S*(*t*) is a Gaussian function with a full-width at half-maximum of 100 fs.

We have also performed calculations for the Floquet band structure of the Hamiltonian (equation (6)) by replacing *S*(*t*)→1 and analysing the thus-obtained time-periodic Hamiltonian.

#### Simulation of trARPES data

From the time-dependent density matrix ***ρ***(**k**, *t*), we computed the time-resolved photoemission spectra through the td-NEGF formalism. As described in refs. ^39,57^, we used the generalized Kadanoff–Baym ansatz that yields the lesser Green’s function from the equation of motion

8 $$[{\rm{i}}{\partial }_{t}-{\bf{H}}({\bf{k}},t)]{{\bf{G}}}^{ < }({\bf{k}},t,{t}^{{\prime} })=0,$$

where **G**^<^(**k**, *t*, *t*) = i**ρ**(**k**, *t*). Using Green’s function, we can then compute the photoemission signal as a function of quasi-momentum **k**, final-state energy *E* and pump–probe delay *τ* as

9 $$\begin{array}{l}I({\bf{k}},E,\tau )\propto {\rm{Im}}\mathop{\sum}\limits_{\alpha {\alpha }^{{\prime} }}{M}_{\alpha }^{* }({\bf{k}},E\,){M}_{{\alpha }^{{\prime} }}({\bf{k}},E\,)\mathop{\int}\nolimits_{0}^{\infty }{\rm{d}}t\mathop{\int}\nolimits_{0}^{t}{\rm{d}}{t}^{{\prime} }\\s(t,\tau )s({t}^{{\prime} },\tau ){{\rm{e}}}^{-{\rm{i}}\varphi ({\bf{k}},t,{t}^{{\prime} })}{G}_{{\alpha }^{{\prime} }\alpha }^{ < }({\bf{k}},{t}^{{\prime} },t)\,.\end{array}$$

In equation (9), *s*(*t*, *τ*) denotes the envelope function of the probe pulse (taken as a Gaussian function with a full-width at half-maximum of 20 fs), whereas the phase factor is defined by

10 $$\varphi ({\bf{k}},t,{t}^{{\prime} })=\mathop{\int}\nolimits_{{\!\!t}^{{\prime} }}^{t}{\rm{d}}\bar{t}\left[{\varepsilon }_{{\rm{f}}}(\bar{t})-{\omega }_{{\rm{pr}}}\right],$$

where *ω*_pr_ is the photon energy of the probe pulse and ${\varepsilon }_{{\rm{f}}}(\bar{t})$ is the light-dressed final-state energy:

11 $${\varepsilon }_{{\rm{f}}}(t)=\frac{{{\bf{p}}}^{2}}{2}-{{\bf{A}}}_{{\rm{eff}}}(t)\cdot {\bf{p}}+\frac{1}{2}{{\bf{A}}}_{{\rm{ef}}\,{\rm{f}}}{(t)}^{2}\,.$$

Here **p**_∥_ = **k**, whereas *p*_⊥_ is determined from **p**^2^/2 = *E*. In our theory, the Volkov effect can be switched off by replacing **A**_eff_→0 in equation (11). Similarly, the case of pure Volkov sidebands can be simulated by replacing **A**_pump_→0 in the time-dependent Hamiltonian (equation (6)).

Our density functional theory code also allows us to compute the photoemission matrix elements

12 $${M}_{\alpha }({\bf{k}},E\,)=\langle {\,\chi }_{{\bf{k}},E}| {{\bf{e}}}_{{\rm{pr}}}\cdot \hat{{\bf{p}}}| {\psi }_{{\bf{k}}\alpha }\rangle \,,$$

where **e**_pr_ denotes the polarization of the *p*-polarized probe pulse and |*χ*_**k**__,__*E*_〉 are the photoelectron states. The predictive power of our method to directly compute the matrix elements (equation (12)) has been confirmed by comparing the calculations to probe the photon-energy-dependent measurements in equilibrium.

Combining the light–matter coupling encoded in equation (6) with the matrix elements (equation (12)) provides us with an ab initio description of time-resolved photoemission (equation (9)).

#### Floquet calculations with scattering

To investigate the role of scattering (as analysed and described in Fig. 5), we use the td-NEGF formalism and include a self-energy that incorporates dissipation and dephasing effects. As a simple model, we assume that the electrons are coupled to a broadband phonon bath, which mimics phenomenological modelling used in previous works^12^ and incorporates the correct physics of the available scattering phase space. The self-energy is constructed as

13 $${{\mathbf{\Sigma }}}^{\gtrless }(t,{t}^{{\prime} })={\rm{i}}{g}^{2}{{\bf{G}}}_{{\rm{loc}}}^{\gtrless }(t,{t}^{{\prime} }){D}^{\gtrless }(t,{t}^{{\prime} })\,.$$

Here

14 $${{\bf{G}}}_{{\rm{loc}}}^{\gtrless }(t,{t}^{{\prime} })=\frac{1}{{N}_{k}}\sum _{{\bf{k}}}{{\bf{G}}}^{\gtrless }({\bf{k}},t,{t}^{{\prime} })$$

denotes the local Green function, whereas *D*^≷^(*t*, *t*′) represents the phonon propagator, defined by the ohmic density of states $B(\omega )=(\omega /{\omega }_{{\rm{c}}}^{2}){{\rm{e}}}^{-\omega /{\omega }_{{\rm{c}}}}$. The cut-off frequency is fixed to *ω*_c_ = 0.02 a.u., which results in a broad *B*(*ω*) value for energies relevant for scattering processes. With the self-energy (equation (13)), we have solved the full two-time Kadanoff–Baym equations for the time-dependent Green functions^58^. To reduce the computational cost, we used the tight-binding model of graphene with the nearest-neighbour hopping here. To isolate the intrinsic linewidth from the finite energy resolution in trARPES, we used a different protocol to extract the Floquet spectra. The electric field was replaced by

15 $${{\bf{E}}}_{{\rm{pump}}}(t)=f(t){\rm{Re}}\left[{{\bf{E}}}_{{\rm{pump}}}{{\rm{e}}}^{-{\rm{i}}\varOmega t}\right]\,,$$

where *f*(*t*) describes a smooth switch-on function with *f*(0) = 0 and *f*(*t*)→1 for *t*→∞. Choosing a sufficiently large simulation time *T*_max_, we define the spectrum as

16 $$A({\bf{k}},\omega )=\frac{1}{2\uppi }{\rm{Im}}{\rm{Tr}}\mathop{\int}\nolimits_{\!0}^{{T}_{\max }}{\rm{d}}t\,{{\bf{G}}}^{ < }({\bf{k}},t,{T}_{\max }){{\rm{e}}}^{{\rm{i}}\omega (t-{T}_{\max })}\,.$$

In the non-interacting limit, *A*(**k**, *ω*) = ∑_*λ*_*n*_*λ*_(**k**)*δ*(*ε*_*λ*_(**k**) – *ω*) traces the Floquet quasi-energy bands *ε*_*λ*_(**k**) (*n*_*λ*_(**k**) is the corresponding weight). In the interacting case, we can study the effects of both broadening and decoherence by inspecting *A*(**k**, *ω*) as a function of the electron–phonon coupling *g*.

To anchor the values of *g* to existing experiments, we first performed a simulation with a short pulse with frequency *Ω* = 1.5 eV, analogous to the experimental protocol in ref. ^20^. We computed the time-dependent current after the pulse, which exhibits gradually damped oscillations due to the decaying coherence. We performed the same simulation for phenomenological dephasing as in ref. ^20^; therefore, we can compare the decay of coherence in the microscopic electron–phonon model to the phenomenological dephasing time *T*_2_.

## Online content

Any methods, additional references, Nature Portfolio reporting summaries, source data, extended data, supplementary information, acknowledgements, peer review information; details of author contributions and competing interests; and statements of data and code availability are available at 10.1038/s41567-025-02889-7.

## Data Availability

The data that support the findings of this study are available from the corresponding authors upon reasonable request.

## Code availability

Program codes for reproducing the theory calculations are available from the corresponding authors upon reasonable request.
